# Impact of short-term exposure to air pollution on natural mortality and vulnerable populations: a multi-city case-crossover analysis in Belgium

**DOI:** 10.1186/s12940-024-01050-w

**Published:** 2024-01-24

**Authors:** Claire Demoury, Raf Aerts, Finaba Berete, Wouter Lefebvre, Arno Pauwels, Charlotte Vanpoucke, Johan Van der Heyden, Eva M. De Clercq

**Affiliations:** 1 Risk and Health Impact Assessment, Sciensano, Brussels, Belgium; 2 Division Ecology, Evolution and Biodiversity Conservation, KU Leuven, Louvain, Belgium; 3 Center for Environmental Sciences, University of Hasselt, Hasselt, Belgium; 4 Health Information, Sciensano, Brussels, Belgium; 5 Flemish Institute for Technological Research (VITO), Mol, Belgium; 6 Belgian Interregional Environment Agency (IRCELINE), Brussels, Belgium

**Keywords:** Air pollution, Natural mortality, Cause-specific mortality, Vulnerability, Effect modification, Preexisting conditions

## Abstract

**Background:**

The adverse effect of air pollution on mortality is well documented worldwide but the identification of more vulnerable populations at higher risk of death is still limited. The aim of this study was to evaluate the association between natural mortality (overall and cause-specific) and short-term exposure to five air pollutants (PM_2.5_, PM_10_, NO_2_, O_3_ and black carbon) and identify potential vulnerable populations in Belgium.

**Methods:**

We used a time-stratified case-crossover design with conditional logistic regressions to assess the relationship between mortality and air pollution in the nine largest Belgian agglomerations. Then, we performed a random-effect meta-analysis of the pooled results and described the global air pollution-mortality association. We carried out stratified analyses by individual characteristics (sex, age, employment, hospitalization days and chronic preexisting health conditions), living environment (levels of population density, built-up areas) and season of death to identify effect modifiers of the association.

**Results:**

The study included 304,754 natural deaths registered between 2010 and 2015. We found percentage increases for overall natural mortality associated with 10 μg/m^3^ increases of air pollution levels of 0.6% (95% CI: 0.2%, 1.0%) for PM_2.5_, 0.4% (0.1%, 0.8%) for PM_10_, 0.5% (-0.2%, 1.1%) for O_3_, 1.0% (0.3%, 1.7%) for NO_2_ and 7.1% (-0.1%, 14.8%) for black carbon. There was also evidence for increases of cardiovascular and respiratory mortality. We did not find effect modification by individual characteristics (sex, age, employment, hospitalization days). However, this study suggested differences in risk of death for people with preexisting conditions (thrombosis, cardiovascular diseases, asthma, diabetes and thyroid affections), season of death (May–September vs October–April) and levels of built-up area in the neighborhood (for NO_2_).

**Conclusions:**

This work provided evidence for the adverse health effects of air pollution and contributed to the identification of specific population groups. These findings can help to better define public-health interventions and prevention strategies.

**Supplementary Information:**

The online version contains supplementary material available at 10.1186/s12940-024-01050-w.

## Background

Air pollution is one of the largest environmental risks to health. According to the World Health Organization, ambient (outdoor) air pollution caused worldwide 4.2 million premature deaths in 2019.

A meta-analysis performed by Orellano et al. [1] reported positive associations between all-cause mortality and short-term exposure to particulate matter with aerodynamic diameters less or equal than 2.5 μm and 10 μm (PM_2.5_, PM_10_) with relative risks (RR) of, respectively, 1.0065 (95% confidence interval (CI): 1.0044, 1.0086) and 1.0041 (95% CI: 1.0034, 1.0049) for an interquartile range increase in pollutant concentration. Increases in nitrogen dioxide (NO_2_) (RR: 1.0072, 95% CI: 1.0059, 1.0085) and ozone (O_3_) (RR: 1.0043, 95% CI: 1.0034, 1.0052) also increased all-cause mortality risk.

PM_2.5_ is composed of different constituents, among which black carbon (BC). BC as other fine particles can easily pass into the blood stream or enter the respiratory system, leading to adverse health effects. While the adverse effects of PM_2.5_ are well documented, less is known about the effects of BC [2]. In 2019, Yang et al. carried out a meta-analysis of the association between fine PM constituents, including BC, and all-cause, cardiovascular and respiratory mortality. Significant associations between all-cause mortality as well as cardiovascular mortality and BC were observed but the meta-analysis was based on no more than four studies [3]. Another recent meta-analysis highlighted the need for more studies considering BC as a separate pollutant of PM_2.5_ [2].

The impact of air pollution on health is documented by studies carried out all over the world [4–6]. However, air pollutants levels highly vary across the regions of the world [7]. Belgium is a small but highly urbanized and industrialized country with a high population density and a dense traffic network [8] in which levels of air pollution above the WHO guidelines are frequently recorded [9, 10]. It is of interest to conduct studies in European countries, because the results of North American or Chinese studies may not be applicable to densely populated countries in Europe, such as Belgium.

Individual or environmental factors can play an important role in modifying the association between air pollution and mortality. However, basic characteristics such as age, sex or socioeconomic status are often the only studied factors [11, 12]. Some studies suggested that health effects can be more severe for people with pre-existing medical conditions. For instance, Bateson and Schwartz found that persons with a history of myocardial infarction as well as those with diabetes had a higher risk of death when exposed to PM_10_ concentrations [13]. In 2016, Alessandrini et al. found increases in natural mortality from PM exposure among people with diabetes and cardiac disorders [14]. However, a recent review conducted by Abed Al Ahad et al. [11] highlighted the lack of studies and a need for further research on individual factors such as pre-existing disease conditions to elucidate their role in the modification of the association between air pollution and mortality, which leads to populations with higher vulnerability to air-pollution.

To fill these gaps, we carried out a multi-city case-crossover analysis at individual level to investigate the impact of short-term exposure to air pollution (PM_2.5_, PM_10_, NO_2_, O_3_ and BC) on daily natural (overall and cause-specific) mortality in nine Belgian agglomerations. The association was estimated for each pollutant in each of the nine agglomerations. Then, the agglomeration-specific estimates were pooled using a random-effect meta-analysis [15] to describe the global association between each air pollutant and mortality. Finally, we used stratified analyses to assess the potential effect modification of this association by individual characteristics (sex, age, employment hospitalization days and chronic preexisting health conditions), living environment (levels of population density, built-up areas) and season of death.

## Methods

### Study area and population

The study area includes the nine largest Belgian municipalities and their agglomerations [16] representing 52.9% of the total Belgian population (11,209,044 inhabitants in 2015) (Table S1). People were included in the study if they were residing in the study area at the time of death and died from natural causes between January 1^st^, 2010 and December 31^st^, 2015 [17].

### Mortality and environmental data

The International Classification of Diseases (10th revision) was used to characterize natural mortality (A00-R99) and cause-specific mortality from cardiovascular diseases (I10-I70), including ischemic heart diseases (IHD) (I20-I25) and cerebrovascular diseases (I60-I69), respiratory diseases (J00-J99) including chronic obstructive pulmonary diseases (COPD) (J40-J44, J47). The date of death, sex, age (5-year age groups), and cause of death were provided by Statbel, the Belgian statistical office.

Environmental exposures were assessed at the geographical coordinates of the residence at the time of death. Daily mean concentrations (in µg/m^3^) of air pollutants (PM_2.5_ (including BC), PM_10_ (including PM_2.5_ and BC), NO_2_, O_3_ and BC) were provided by the Belgian Interregional Environment Agency and estimated by RIO-IFDM models with 100 m spatial resolution [18]. The RIO-IFDM model consists of the RIO background model at 4 km × 4 km, which is a land use regression model interpolating concentration measurements, combined with the IFDM bi-Gaussian dispersion model which calculates the dispersion of road traffic, shipping traffic and large industrial point sources. Other sources such as residential heating or agricultural emissions are included in the RIO background model through the measurements. Finally, we also obtained information on daily mean temperature and relative humidity by the Royal Meteorological Institute of Belgium [17, 19].

### Additional data for subgroups analyses

We used indicators of “pseudopathologies” (thrombosis, cardiovascular diseases (CVD), COPD, asthma, diabetes, psychoses, and thyroid affections), based on reimbursed medication dispensed in pharmacies (except those dispensed in hospital settings and some nursing homes) as proxy to determine the presence of preexisting chronic diseases in people. Data on reimbursed medication were obtained from the InterMutualistic Agency (IMA), an organization which hosts exhaustive data from the mandatory Belgian health insurance. People were considered as having a specific pseudopathology if they had received prescriptions for more than 90 defined daily doses (DDD) of drugs belonging to specific anatomical therapeutic chemicals (ATC) categories (WHO Collaborating Centre for Drug Statistics Methodology) in the 12 months preceding death. Since DDD are defined for adults, we only considered subjects older than 20 years (Table S2). From IMA databases, we also obtained information on hospitalization days (cumulative total above 120 days in the year preceding death) and employment of people (yes/no) at the end of the calendar year preceding death.

Individual (pseudonymized) data were linked using the Belgian national register number, which allows to identify all the Belgian residents.

Finally, we obtained the population density of the census tract of residence from Statbel and we defined an indicator of built-up area calculated as the percentage of non-vegetated surface for a 1-km buffer around the residence (MODIS/Terra Vegetation Continuous Fields, 250 m spatial resolution) [20].

### Statistical analyses

A time-stratified case-crossover design was used to assess the association between mortality and each air pollutant, separately. This design was proposed by Maclure et al. to assess the effect of a transient exposure on acute health outcomes [21]. This design has the advantage of controlling for variables which do not vary on a short time (sex, age or socioeconomic status for instance). In such a design, a case day is defined by the date of death and control days are defined in a short period before or after the case days. In a time-stratified approach, control days are selected from the same day of the week, month and year as the case day [22], controlling for seasonality and time trends.

### Association between air pollutants and mortality

We followed a two-stage approach: first using conditional logistic regressions, we assessed the association between air pollutants and mortality in the nine agglomerations. To determine an appropriate lag time between exposure and the outcome for each pollutant, we compared a variety of single and cumulated lag days, up to 15 days, using the distributed lag linear models (DLM) proposed by Gasparrini et al. [23]. The lag which yielded the minimal summed Akaike Information Criterion (AIC) over the nine agglomerations was selected for the analyses [23]. To control for potential confounding, we adjusted in the conditional logistic regressions for daily mean temperature and relative humidity with 0–3 days moving averages using natural cubic splines with 3 degrees of freedom (df).

In a second step, a random-effect meta-analysis [15] was performed to pool the agglomeration-specific estimates and describe the global association between each air pollutant and mortality. Cochran’s Q test and the I-squared statistics were used to examine the between agglomeration heterogeneity.

Odds ratios resulting from the logistic regressions were converted into percentage changes for ease of interpretation.

Additionally, using the model specification aforementioned, we introduced air pollutants with a natural cubic spline function with 3 df (using distributed lag non-linear models [23]) to visualize and detect possible departure from linearity of the air pollutants-mortality relationships.

The packages survival, dlnm and mvmeta of the R software (R Foundation for Statistical Computing, Vienna, Austria) were used to perform the analyses.

### Subgroups analyses and effect modification

To detect potential effect modification of the association between air pollutants and all-cause mortality, stratified analyses were carried out for individual characteristics (sex, age, employment status, hospitalization days, pseudopathologies), environmental variables (population density, built-up area) and season of death (May–September vs October–April)).

Effect modification was evaluated using the Z-test and comparing Z to the standard normal distribution [24]:

$$Z=\frac{{\beta }_{1}-{\beta }_{2}}{\sqrt{{SE\left({\beta }_{1}\right)}^{2}+{SE\left({\beta }_{2}\right)}^{2}}}$$

β_1_ and β_2_ are the effect estimates in two subgroups, SE(β_1_) and SE(β_2_) their respective standard errors.

### Sensitivity analyses

To examine the robustness of our findings, we conducted several sensitivity analyses. First, we performed two-pollutant models by simultaneously introducing in the models pollutants that are not highly correlated (Pearson’s correlation coefficient < 0.8). Second, for each pollutant, instead of the lags minimizing the AICs, we performed analyses considering cumulated lags 1) 0 to 1 days and 2) 0 to 5 days. Finally, for meteorological variables (mean temperature and relative humidity), we 1) controlled 0–1 days moving averages instead of 0–3 days, 2) varied the df values of the natural cubic splines from 3 to 6.

## Results

Between 2010 and 2015, 307,859 natural deaths were registered in the study area of which 307,490 geocoding of the address were possible. People geocoded with less precision (extrapolation of the street number) or people living < 200 m from a tunnel exit were excluded from the analyses (*N* = 2,736). The latter showed unrealistically high values of exposure. The population hence includes 304,754 people and IMA information was available for 300,492 of them. Deaths from cardiovascular and respiratory causes represented 29.7% and 11.2% of cases, respectively (Table 1).

**Table 1:** Percentage changes for all-causes and cause-specific mortality associated with 10 μg/m^3^ air pollutants increase

Deaths	n (%)	PM_2.5_	PM_10_	O_3_	NO_2_	Black carbon
All-causes	304,754 (100)	0.6 (0.2,1.0)^a^	0.4 (0.1,0.8)^a^	0.5 (-0.2,1.1)	1.0 (0.3,1.7)^a^	7.1 (-0.1,14.8)
Cardiovascular	90,480 (29.7)	0.5 (-0.3,1.4)	0.3 (-0.5,1.1)	0.4 (-0.4,1.3)	1.2 (-0.4,2.8)	8.0 (-6.0,24.1)
IHD	25,868 (8.5)	0.4 (-0.9,1.8)	0.2 (-1.0,1.3)	1.6 (0.5,2.8)^a^	-0.1 (-2.6,2.6)	0.4 (-17.4,22.0)
Cerebrovascular	21,522 (7.1)	-0.1 (-1.5,1.4)	0.0 (-1.3,1.3)	-0.2 (-1.9,1.7)	1.6 (-0.2,3.3)	5.4 (-11.4,25.4)
Respiratory	34,162 (11.2)	0.6 (-0.5,1.7)	0.3 (-0.6,1.4)	0.4 (-1.1,1.9)	0.7 (-0.7,2.0)	10.5 (-3.4,26.5)
COPD	13,794 (4.5)	0.4 (-1.4,2.2)	0.3 (-1.2,1.9)	1.7 (-0.7,4.1)	-0.6 (-2.7,1.5)	7.9 (-12.9,33.6)
Other natural	180,112 (59.1)	0.8 (0.2,1.3)^a^	0.5 (0.1,1.0)^a^	0.6 (-0.1,1.2)	0.9 (0.3,1.5)^a^	5.5 (-0.7,12.1)

*n*: number of deaths, *IHD* Ischemic heart diseases, *COPD* Chronic obstructive pulmonary

Percentage changes were assessed on different lag days: lag 0 for O_3_, lag 7 for PM_2.5_, PM_10_, NO_2_ and black carbon

^a^significant percentage increases at 5% level

The average daily concentrations for PM_2.5_, PM_10_, O_3_, NO_2_ and BC were 15.3 µg/m^3^ (sd: 11.9), 22.2 µg/m^3^ (sd: 13.4), 39.3 µg/m^3^ (sd: 19.4), 25.8 µg/m^3^ (sd: 13.2) and 1.6 µg/m^3^ (sd: 1.1) respectively. There were no large differences in concentrations between agglomerations (< 10 µg/m^3^) except for one agglomeration (Antwerp) showing higher levels of NO_2_ concentrations (Table S3). Pollutant concentrations were similar in subgroups of populations stratified by individual characteristics and pseudopathologies (Table S4). There were, however, higher levels of PM_2.5_, PM_10_, NO_2_ and BC and lower levels of O_3_ concentrations in densely populated and highly built up areas as well as during the cold season (Table S4).

**Fig. 1:**
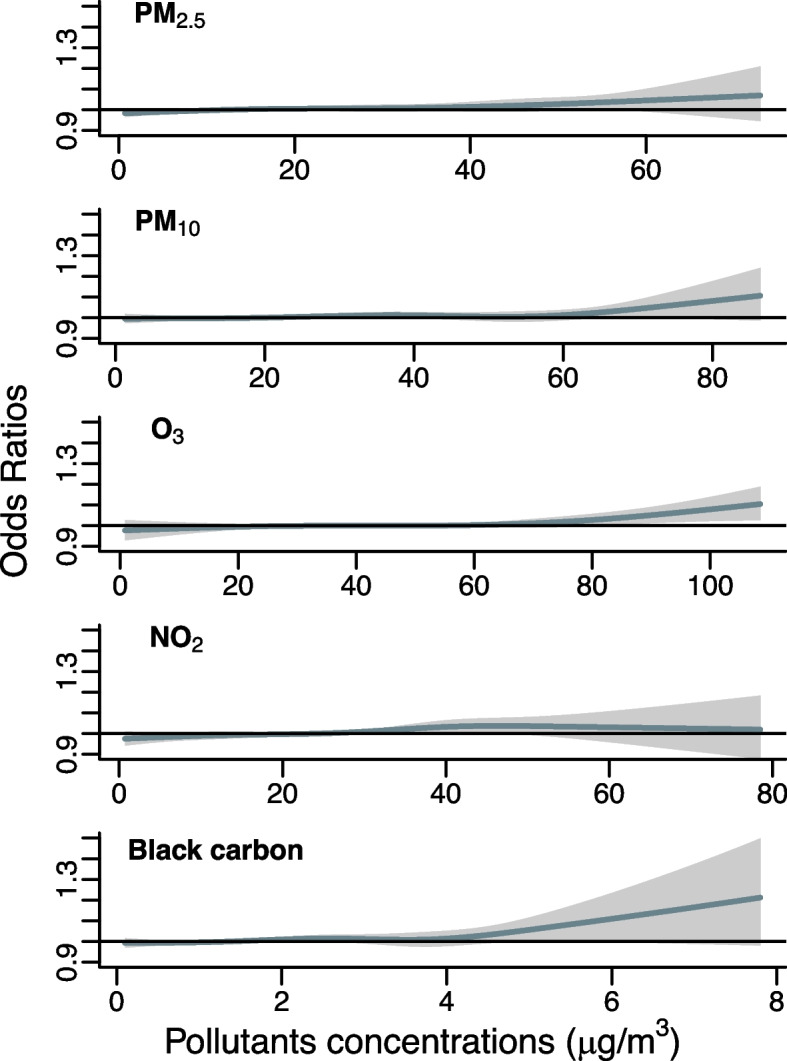
Air pollutants-mortality curves modeled using natural cubic spline functions BC: black carbon; Percentage changes were assessed on different lag days: lag 0 for O_3_, lag 7 for PM_2.5_, PM_10_, NO_2_ and BC

The pooled associations between air pollutants and mortality for PM, NO_2_ and BC showed no between agglomeration heterogeneity (I-squared range: 0.01%-18.2%, Cochran Q-test p-values range: 0.18–0.77) and moderate heterogeneity for O_3_ (I-squared: 48.9%, Cochran Q-test *p*-value: 0.001) (Table S5). We did not observe strong departure from linearity when modeling air pollutants-mortality curves with natural cubic spline functions (Fig. 1, Figure S1-S5) and thus modeled air pollutants as linear variables in further analyses. For PM, O_3_ and NO_2_, no obvious thresholds were observed, indicating positive linear associations. For BC, an increase in risk appeared at high concentrations (> 4 µg/m^3^) but the curve showed high uncertainties for these levels of concentrations. The minimum AICs were obtained for single lag days, at lag 0 for O_3_ and at lag 7 for the four other pollutants. The respective percentage changes for all-causes mortality associated with 10 μg/m^3^ increase in pollutants were 0.6% (95% CI: 0.2%, 1.0%) for PM_2.5_, 0.4% (0.1%, 0.8%) for PM_10_, 0.5% (-0.2%, 1.1%) for O_3_, 1.0% (0.3%, 1.7%) for NO_2_ and 7.1% (-0.1%, 14.8%) for BC (Table 1). We also observed percentage increases for increases in all pollutants concentrations for all-cardiovascular, all-respiratory as well as other natural mortality. Regarding more specific subgroups (IHD, cerebrovascular and COPD mortality), we found a significant increase of 1.6% (95% CI: 0.5%, 2.8%) in IHD mortality for an increase in O_3_ concentration (Table 1).

**Fig. 2:**
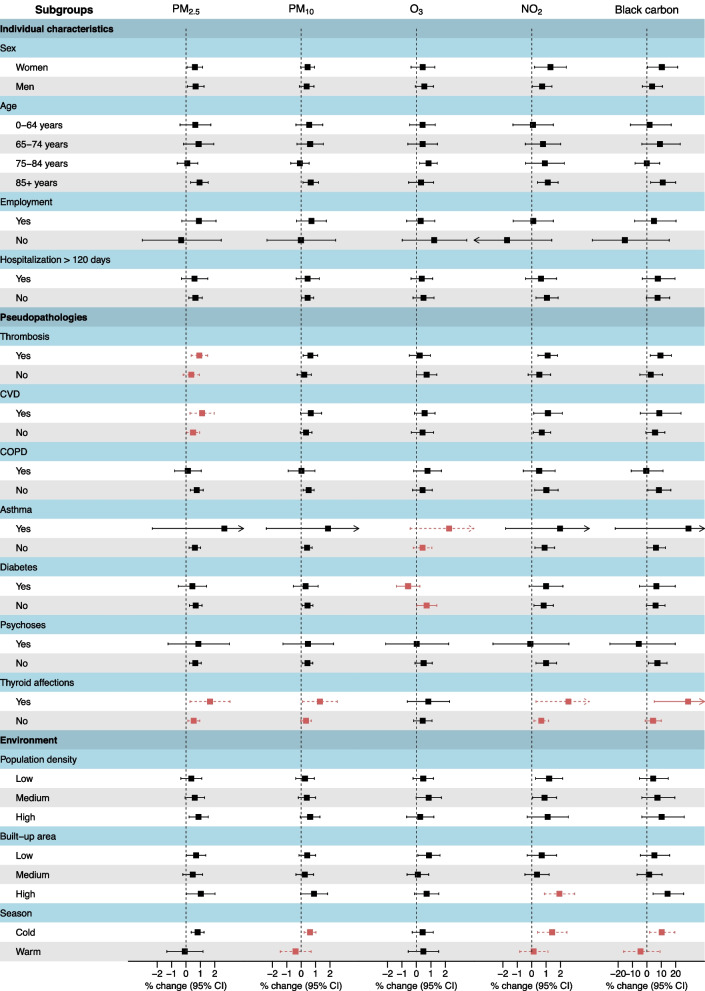
Percentage changes for all-causes mortality associated with air pollutants increase by subgroups of population BC: black carbon; CVD: cardiovascular diseases; COPD: chronic obstructive pulmonary diseases Red: significant effect modification (*p*<0.05); Dashed red: suggestion of effect modification (*p* between 0.05 and 0.20) [25]. *P*-values for effect modification were assessed by Z-tests, which examined the statistical significance of the effect differences between different subgroups; for variables containing more than two categories, p was calculated by comparing the estimate of the specified category with the first category [26] Percentage changes were assessed on different lag days: lag 0 for O_3_, lag 7 for PM_2.5_, PM_10_, NO_2_ and BC

Stratified analyses did not show effect modification of the association between most of the individual characteristics (sex, age, employment and hospitalization days) and air pollutants (*p* > 0.05) (Fig. 2). Regarding preexisting conditions, there was, however, a significantly lower risk of death in people with diabetes for O_3_ (*p* = 0.02). We also observed a significant effect modification for thyroid affections and BC (*p* = 0.049) as well as suggestion of effect modification (*p* between 0.05 and 0.20 [25]) for PM_2.5_, PM_10_, and NO_2_. In addition, there was a suggestion of a higher risk of death in people with thrombosis or CVD for an increase in PM_2.5_ concentrations. Finally, there was a suggestion of effect modification for asthma and O_3_. It can be noted that people with asthma were at higher risk for all pollutants and that this group represents few people (Table S4). Concerning the environmental variables, a higher risk was suggested when NO_2_ increased for people living in highly built-up areas. There was also a suggestion of a higher risk during the cold season (October–April) when PM_10_, NO_2_ and BC increased.

Two-pollutants models were run for all pairs of pollutants except for the PM_2.5_ and PM_10_ pair, and the NO_2_ and BC pair because Pearson’s correlation coefficients were higher than 0.8 (Table S6). Adjustment for O_3_ did not change the results and O_3_ estimates were not modified by adjustment for the other pollutants (Table 2). We observed a decrease in PM_2.5_ estimate after adjustment for NO_2_ but not after adjustment for BC. There was also a decrease in PM_10_ estimates with adjustment for NO_2_ and to a lesser extent for BC. NO_2_ and BC estimates were attenuated when models were adjusted for PM_2.5_ and to a lesser degree for PM_10_.

**Table 2:** Percentage changes in all-causes mortality associated with 10 μg/m^3^ air pollutants increase in two-pollutants models

	PM_2.5_	PM_10_	O_3_	NO_2_	Black carbon
*Adjustment for:*					
PM_2.5_ (lag 7)	-	-	0.4 (-0.2,1.1)	0.7 (-0.1,1.4)	2.3 (-6.4,11.9)
PM_10_ (lag 7)	-	-	0.4 (-0.2,1.1)	0.9 ( 0.0,1.9)	5.2 (-4.6,16.1)
O_3_ (lag 0)	0.6 ( 0.2,1.0)	0.4 ( 0.0,0.7)	-	1.0 ( 0.3,1.7)	7.1 (-0.5,15.3)
NO_2_ (lag 7)	0.3 (-0.2,0.8)	0.0 (-0.4,0.5)	0.4 (-0.2,1.1)	-	-
Black carbon (lag 7)	0.6 ( 0.0,1.1)	0.2 (-0.3,0.7)	0.4 (-0.2,1.1)	-	-

Two-pollutants models were run for pairs of pollutants with Pearson’s correlation coefficient < 0.8 (i.e. excluding the PM_2.5_ and PM_10_ pair and the NO_2_ and black carbon pair (Table S6))

Results were robust to changes in the lags specification when using cumulated lags from 0 to 5 days, but for shorter lags of 0 to 1 days, percentage changes became non-significant for all the pollutants. Results were also robust to changes in the meteorological variables specification: increasing the number of df from 3 to 6 in natural cubic splines as well as considering 0–1 days moving averages gave similar estimates, although the percentage increase for BC became significant when changing the definition of the moving average (Table S7).

## Discussion

In this study, we found evidence for increases in all-cause, cardiovascular and respiratory mortality with increases in PM_2.5_, PM_10_, NO_2_, O_3_ and BC concentrations, although the increases were significant for all-cause mortality and PM_2.5_, PM_10_, NO_2_ only. There was also evidence for a significant increase in IHD mortality with an increase in O_3_ concentration. We did not find effect modification of the association between mortality and any of the air pollutants by individual characteristics (sex, age, employment, hospitalization days). However, this study suggested differences in risks for people with chronic preexisting conditions (thrombosis, cardiovascular diseases, asthma, diabetes and thyroid affections). Finally, our results also suggest effect modification by levels of built-up area (for NO_2_) and season of death.

In our investigation of effect modification of the air pollution-related mortality association by pseudopathologies, there was suggestion for a higher vulnerability to PM_2.5_ in people with thrombosis and cardiovascular diseases. People with thyroid affections seemed to be more vulnerable to PM, NO_2_ and BC. These findings are in line with other studies reporting a higher vulnerability to PM_10_ in people with pre-existing conditions such as myocardial infarction and congestive heart failure [13], hypertension and chronic obstructive pulmonary disease [27], pneumonia, stroke and heart failure [28]. Some cardiovascular conditions (i.e., ischemic heart disease, pulmonary circulation impairment, heart conduction disorders, heart failure) were also associated with a greater vulnerability to NO_2_ [29]. A recent meta-analysis reported an increased risk of death from heart failure associated with short-term exposure to PM and NO_2_ as well as sulphur dioxide and carbon monoxide, but not O_3_ [30]. Our investigation also suggested a higher vulnerability to O_3_ in people with asthma. Interestingly, the risk was also higher for the four other pollutants even far from significance. Asthma has often been found to enhance vulnerability to air pollution [31]. The small number of people having asthma in our study, these people representing only 1.5% of our population, can explain the large uncertainties in the effect estimates and the absence of significant effect modification. In this study, we also reported lower risks of death in people with diabetes for increased levels of O_3_, which differ from the findings of Stafoggia et al. 2010 in a study conducted in 10 Italian cities [32]. Another study reported positive associations between type 2 diabetes mortality and PM and NO_2_ but O_3_ was not investigated [33]. Diabetes has actually been more often reported to increase vulnerability to PM-related mortality [13, 14, 27, 28].

Our study suggested a higher risk of death associated with NO_2_ in highly built-up areas in comparison to less built-up areas. NO_2_ being a highly traffic-related pollutant, traffic load and street canyon effects can explain higher concentrations of NO_2_ in urban areas and an increase in the risk of death. By reducing NO_2_ levels, higher levels of greenness in less built-up areas might also explain these findings [34]. Besides, there was no suggestion of higher risks in highly built-up areas for other pollutants nor in densely populated areas, suggesting that people living in rural areas are also subject to the adverse effects of air pollution. Finally, our study suggested higher effects of PM, NO_2_ and BC pollutants on mortality during the cold season. Such findings have been observed elsewhere [35, 36] but on the contrary, higher risks during the warm season were also observed in other studies [5, 37, 38]. In this study, we reported higher concentrations of PM, NO_2_ and BC during the cold season compared to the warm season. Seasonal variations in pollutants levels, different according to the location, could explain the discrepancy between results.

Using objective measures of exposure provided by modeled concentrations of air pollutants, we observed increases in mortality risks with levels of air pollution but as reported by Kangas et al. for the Brussels-Capital Region (Belgium), an increase in mortality has also been linked to increased subjective measures of air pollution (consisting in self-reported perceived air quality) [39].

In two-pollutants models, although all associations remained positive with adjustment for other pollutants, we observed some attenuated associations: adjustment for NO_2_ decreased the PM-mortality association and the BC–mortality association decreased after adjusting for PM. These findings are in line with other studies showing lower effects of PM with adjustment for NO_2_ [40–42] and slightly BC estimates when adjusting for PM [3]. Nevertheless, our study supports the need for additional studies aimed at better differentiating the effects of individual pollutants on mortality from the associations due to pollutants acting as proxies of others.

This study has several strengths and limitations. First, we obtained individual data for more than half of the population of Belgium. Then, we used the most accurate exposure assessment by linking high-resolution models to the exact geographical coordinates of the address of residence to estimate their exposure to air pollution. In this way, the chance of missing local phenomena (i.e. very small areas recording high values of pollutants concentrations) was reduced. The possibility of exposure misclassification was thus reduced in this study by using geographic point coordinates rather than administrative unit polygons [43]. However, exposure was assessed at the address of residence at the time of death because the exact address of the place of death, often in the hospital, was not available. Exposure to air pollution in the few days preceding death can differ in both places. In this study, we performed stratified analyses with variables going from basic individual characteristics (age, sex) to environmental variables and pre-existing chronic health conditions, which were scarcely investigated in air pollution studies. It is however difficult to determine the chronic disease status of individuals based on medication data because the type and volume of medication is not always specific enough to distinguish between chronic diseases. Additionally, we cannot exclude that a proportion of the population with chronic diseases did not take the prescribed medications but also that some people who took the medication used to define a chronic disease used this medication for other reasons. More generally, a clear definition of chronic health conditions is missing for many chronic diseases so that clearly defining groups of chronic diseases remains difficult [44]. However, Berete et al. showed that the pseudopathology definitions used in this study correctly identify people suffering from several important diseases (cardiovascular diseases, diabetes, thyroid disorders) [45].

## Conclusions

This study provided evidence for an increased risk of death associated with air pollution concentrations in Belgium. By identifying more vulnerable populations, the study also contributed to the identification of specific population groups with a higher risk of adverse health effects of air pollution. These findings can help to better define targeted health policies and prevention initiatives to reduce the health impact of air pollution.

### Supplementary Information

**Additional file 1: Table S1. **Summary statistics for population and mortality by agglomeration. **Table S2.** Algorithms defining the pseudopathologies. **Table S3.** Pollutants averaged (2010-2015) daily concentrations (µg/m^3^) by agglomeration. **Table S4.** Summary statistics for air pollutants by subgroups, 2010-2015. **Table S5.** I-square statistic and p-values for Cochran Q-test for heterogeneity. **Table S6.** Pearson correlations between pollutants. **Table S7.** Percentage changes and 95% confidence intervals associated with 10 μg/m^3^ increase in PM_2.5_, PM_10_, O_3_, NO_2_ and black carbon, 2010-2015 in sensitivity analyses. **Figure S1.** Agglomeration-specific and pooled PM_2.5_-mortality relationships. **Figure S2.** Agglomeration-specific and pooled PM_10_-mortality relationships. **Figure S3.** Agglomeration-specific and pooled O_3_-mortality relationships. **Figure S4.** Agglomeration-specific and pooled NO_2_ -mortality relationships. **Figure S5.** Agglomeration-specific and pooled black carbon-mortality relationships.

## Data Availability

The data result from the linkage of administrative databases and has been collected in the framework of the HEASP project (Belgian Statistical Office—approval n°2018/014 from the 19^th^ of June, 2018 and Sectoral Committee on Social Security and Health—approval n°18/080 from the 5^th^ of June, 2018). Restrictions apply to the availability of these data, and so are not publicly available.

## Abbreviations

AIC: Akaike Information Criterion
BC: Black carbon
CI: Confidence interval
COPD: Chronic obstructive pulmonary diseases
CVD: Cardiovascular diseases
DLM: Distributed lag linear models
IHD: Ischemic heart disease
IMA: InterMutualistic Agency
NO_2_: Nitrogen dioxide
O_3_: Ozone
PM_2.5_: Particulate matter with aerodynamic diameters less or equal than 2.5 μm
PM_10_: Particulate matter with aerodynamic diameters less or equal than 10 μm
RR: Relative risk
