# Supplementary material for: Impact of short-term exposure to air pollution on natural mortality and vulnerable populations: a multi-city case-crossover analysis in Belgium
Source: Environ Health. 2024 Jan 24;23:11. doi: 10.1186/s12940-024-01050-w (PMC10809644; doi:10.1186/s12940-024-01050-w)
Supplement: Supplementary file 1 — Additional file 1: Table S1. Summary statistics for population and mortality by agglomeration. Table S2. Algorithms defining the pseudopathologies. Table S3. Pollutants averaged (2010-2015) daily concentrations (µg/m3) by agglomeration. Table S4. Summary statistics for air pollutants by subgroups, 2010-2015. Table S5. I-square statistic and p-values for Cochran Q-test for heterogeneity. Table S6. Pearson correlations between pollutants. Table S7. Percentage changes and 95% confidence intervals associated with 10 μg/m3 increase in PM2.5, PM10, O3, NO2 and black carbon, 2010-2015 in sensitivity analyses. Figure S1. Agglomeration-specific and pooled PM2.5-mortality relationships. Figure S2. Agglomeration-specific and pooled PM10-mortality relationships. Figure S3. Agglomeration-specific and pooled O3-mortality relationships. Figure S4. Agglomeration-specific and pooled NO2 -mortality relationships. Figure S5. Agglomeration-specific and pooled black carbon-mortality relationships. [file 12940_2024_1050_MOESM1_ESM.docx]

**Impact of short-term exposure to air pollution on natural mortality and vulnerable populations: a multi-city case-crossover analysis in Belgium**

**Supplemental Materials**

[Table S1. Summary statistics for population and mortality by agglomeration 2](#_Toc152751855)

[Table S2. Algorithms defining the pseudopathologies 2](#_Toc152751856)

[Table S3. Pollutants averaged (2010-2015) daily concentrations (µg/m^3^) by agglomeration 3](#_Toc152751857)

[Table S4. Summary statistics for air pollutants by subgroups, 2010-2015 4](#_Toc152751858)

[Table S5. I-square statistic and p-values for Cochran Q-test for heterogeneity 6](#_Toc152751859)

[Table S6. Pearson correlations between pollutants 6](#_Toc152751860)

[Table S7. Percentage changes and 95% confidence intervals associated with 10 μg/m^3^ increase in PM_2.5_, PM_10_, O_3_, NO_2_ and black carbon, 2010-2015 in sensitivity analyses. 6](#_Toc152751861)

[Figure S1. Agglomeration-specific and pooled PM_2.5_-mortality relationships 7](#_Toc152751862)

[Figure S2. Agglomeration-specific and pooled PM_10_-mortality relationships 8](#_Toc152751863)

[Figure S3. Agglomeration-specific and pooled O_3_-mortality relationships 9](#_Toc152751864)

[Figure S4. Agglomeration-specific and pooled NO_2_ -mortality relationships 10](#_Toc152751865)

[Figure S5. Agglomeration-specific and pooled black carbon-mortality relationships 11](#_Toc152751866)

# Table S1. Summary statistics for population and mortality by agglomeration

|  | Municipalities | Population 2015 | Deaths 2010-2015 |
| --- | --- | --- | --- |
|  | n (%) | n (%) | n (%) |
| Antwerp | 30 (13.8) | 1,027,342 (17.3) | 53,335 (17.5) |
| Bruges | 10 (4.6) | 280,153 (4.7) | 16,030 (5.3) |
| Brussels | 62 (28.4) | 1,997,349 (33.7) | 87,654 (28.8) |
| Charleroi | 14 (6.4) | 429,854 (7.3) | 26,532 (8.7) |
| Ghent | 22 (10.1) | 557,453 (9.4) | 28,657 (9.4) |
| Leuven | 27 (12.4) | 446,183 (7.5) | 22,821 (7.5) |
| Liège | 24 (11.0) | 618,887 (10.4) | 37,298 (12.2) |
| Mons | 13 (6.0) | 257,804 (4.3) | 15,876 (5.2) |
| Namur | 16 (7.3) | 311,684 (5.3) | 16,551 (5.4) |
| All | 218 | 5,926,709 (100) | 304,754 (100) |

# Table S2. Algorithms defining the pseudopathologies

| Pseudopathologies | ATC and conditions on age |
| --- | --- |
| Thrombosis | B01A |
| CVD | C01 |
| COPD | R03BB, R03DA04, R03A & age > 50, R03BA & age > 50 |
| Asthma | R03DC01, R03DC03, R03DX05, R03A & age <= 50, R03BA & age <= 50 |
| Diabetes | A10A, A10B |
| Psychoses | N05AA, N05AB, N05AC, N05AD, N05AE, N05AF, N05AG, N05AH, N05AN, N05AX, N07XX06 |
| Thyroid affections | H03AA |

ATC: Anatomical Therapeutic Chemical ; CVD: cardiovascular diseases ; COPD: chronic obstructive pulmonary diseases

# Table S3. Pollutants averaged (2010-2015) daily concentrations (µg/m^3^) by agglomeration

| **Agglomerations** | **mean ± sd** | **min** | **p25** | **median** | **p75** | **max** |
| --- | --- | --- | --- | --- | --- | --- |
| ***PM_2.5_*** |  |  |  |  |  |  |
| Antwerp | 16.9 ± 12.1 | 2.0 | 8.6 | 13.1 | 21.5 | 73.1 |
| Bruges | 15.4 ± 11.9 | 2.2 | 7.2 | 11.3 | 19.1 | 70.3 |
| Brussels | 16.6 ± 11.7 | 1.0 | 8.5 | 13.2 | 21.2 | 71.0 |
| Charleroi | 13.4 ± 11.7 | 0.8 | 4.8 | 9.5 | 18.4 | 62.9 |
| Ghent | 16.3 ± 12.4 | 1.8 | 7.6 | 12.2 | 20.7 | 72.7 |
| Leuven | 14.7 ± 11.4 | 1.2 | 6.8 | 11.1 | 18.8 | 68.3 |
| Liège | 13.2 ± 11.7 | 0.7 | 4.9 | 9.6 | 18.0 | 66.5 |
| Mons | 12.7 ± 10.9 | 0.7 | 4.7 | 9.3 | 17.1 | 59.8 |
| Namur | 12.2 ± 11.1 | 0.8 | 4.2 | 8.7 | 16.9 | 61.5 |
| All | 15.3 ± 11.9 | 0.7 | 7.1 | 11.8 | 19.9 | 73.1 |
| ***PM_10_*** |  |  |  |  |  |  |
| Antwerp | 25.0 ± 13.6 | 4.2 | 15.8 | 21.1 | 30.2 | 86.6 |
| Bruges | 21.3 ± 12.4 | 3.5 | 13.1 | 17.7 | 25.7 | 74.7 |
| Brussels | 22.6 ± 13.2 | 3.0 | 13.3 | 19.0 | 28.2 | 81.4 |
| Charleroi | 20.7 ± 13.6 | 3.4 | 10.5 | 16.6 | 26.7 | 78.0 |
| Ghent | 24.1 ± 13.5 | 4.9 | 14.7 | 20.3 | 29.2 | 82.8 |
| Leuven | 20.4 ± 12.8 | 3.4 | 11.6 | 16.6 | 25.1 | 80.5 |
| Liège | 20.8 ± 14.0 | 1.3 | 10.7 | 16.5 | 26.8 | 84.6 |
| Mons | 19.4 ± 12.3 | 4.0 | 10.2 | 15.8 | 24.9 | 68.5 |
| Namur | 18.5 ± 12.7 | 2.3 | 9.1 | 14.7 | 23.7 | 73.8 |
| All | 22.2 ± 13.4 | 1.3 | 12.8 | 18.5 | 27.7 | 86.6 |
| ***O_3_*** |  |  |  |  |  |  |
| Antwerp | 34.7 ± 19.5 | 0.8 | 19.4 | 34.1 | 48.1 | 95.4 |
| Bruges | 42.7 ± 18.4 | 0.8 | 30.0 | 44.4 | 56.1 | 87.6 |
| Brussels | 38.2 ± 19.3 | 1.0 | 24.0 | 38.1 | 51.3 | 98.3 |
| Charleroi | 38.8 ± 18.7 | 1.0 | 26.1 | 38.1 | 50.7 | 100.3 |
| Ghent | 40.4 ± 18.8 | 1.0 | 26.5 | 41.2 | 54.1 | 92.0 |
| Leuven | 43.0 ± 19.8 | 1.6 | 29.1 | 42.9 | 56.1 | 107.6 |
| Liège | 42.9 ± 20.0 | 1.2 | 29.1 | 42.8 | 55.5 | 108.6 |
| Mons | 37.5 ± 17.6 | 1.0 | 24.9 | 37.2 | 49.4 | 90.7 |
| Namur | 43.8 ± 19.0 | 1.2 | 31.2 | 44.0 | 56.0 | 102.9 |
| All | 39.3 ± 19.4 | 0.8 | 25.1 | 39.3 | 52.5 | 108.6 |
| ***NO_2_*** |  |  |  |  |  |  |
| Antwerp | 31.3 ± 14.0 | 1.6 | 20.9 | 29.4 | 39.8 | 78.5 |
| Bruges | 20.9 ± 11.1 | 1.0 | 12.6 | 18.9 | 27.4 | 57.5 |
| Brussels | 29.0 ± 13.9 | 1.5 | 18.5 | 26.9 | 37.4 | 76.0 |
| Charleroi | 24.1 ± 11.3 | 1.7 | 15.6 | 22.4 | 30.8 | 61.5 |
| Ghent | 23.5 ± 12.6 | 0.7 | 13.9 | 21.5 | 31.1 | 65.3 |
| Leuven | 20.6 ± 10.2 | 1.2 | 12.8 | 19.0 | 26.6 | 54.2 |
| Liège | 23.7 ± 11.9 | 1.0 | 14.7 | 21.5 | 30.6 | 65.2 |
| Mons | 21.0 ± 10.1 | 1.0 | 13.4 | 19.2 | 26.9 | 56.1 |
| Namur | 19.1 ± 10.0 | 0.8 | 11.6 | 17.2 | 24.7 | 53.7 |
| All | 25.8 ± 13.2 | 0.7 | 15.8 | 23.5 | 33.5 | 78.5 |
| ***BC*** |  |  |  |  |  |  |
| Antwerp | 1.9 ± 1.3 | 0.1 | 1.0 | 1.5 | 2.4 | 7.9 |
| Bruges | 1.4 ± 1.0 | 0.1 | 0.7 | 1.1 | 1.8 | 6.1 |
| Brussels | 1.8 ± 1.2 | 0.1 | 1.0 | 1.5 | 2.3 | 7.2 |
| Charleroi | 1.5 ± 1.0 | 0.1 | 0.8 | 1.2 | 1.9 | 6.4 |
| Ghent | 1.6 ± 1.1 | 0.1 | 0.8 | 1.3 | 2.0 | 6.4 |
| Leuven | 1.3 ± 0.8 | 0.1 | 0.7 | 1.1 | 1.6 | 5.1 |
| Liège | 1.7 ± 1.1 | 0.1 | 0.9 | 1.4 | 2.1 | 6.6 |
| Mons | 1.3 ± 0.9 | 0.1 | 0.7 | 1.1 | 1.7 | 5.6 |
| Namur | 1.2 ± 0.8 | 0.1 | 0.6 | 1.0 | 1.5 | 5.0 |
| All | 1.6 ± 1.1 | 0.1 | 0.8 | 1.3 | 2.1 | 7.9 |

n: number of deaths ; sd: standard deviation ; min: minimum ; p25 and p75: percentiles of the pollutants concentrations distribution ; max: maximum

# Table S4. Summary statistics for air pollutants by subgroups, 2010-2015

|  |  |  | PM_2.5_ | PM_10_ | O_3_ | NO_2_ | BC |
| --- | --- | --- | --- | --- | --- | --- | --- |
|  |  | n (%) | mean ± sd | | | | |
| All |  |  |  |  |  |  |  |
| *Individual characteristics* |  |  |  |  |  |  |  |
| Sex | Women | 160,131 (52.5) | 15.3 ± 11.9 | 22.2 ± 13.5 | 39.2 ± 19.5 | 25.9 ± 13.2 | 1.6 ± 1.1 |
|  | Men | 144,623 (47.5) | 15.2 ± 11.9 | 22.1 ± 13.4 | 39.3 ± 19.4 | 25.7 ± 13.2 | 1.6 ± 1.1 |
| Age | 0-64 years | 43,300 (14.2) | 15.0 ± 11.8 | 21.9 ± 13.4 | 39.0 ± 19.3 | 25.9 ± 13.2 | 1.7 ± 1.1 |
|  | 65-74 years | 44,714 (14.7) | 15.1 ± 11.7 | 22.0 ± 13.3 | 39.4 ± 19.4 | 25.6 ± 13.2 | 1.6 ± 1.1 |
|  | 75-84 years | 91,510 (30.0) | 15.2 ± 11.8 | 22.1 ± 13.4 | 39.4 ± 19.5 | 25.6 ± 13.2 | 1.6 ± 1.1 |
|  | 85+ years | 125,230 (41.1) | 15.5 ± 12.0 | 22.4 ± 13.5 | 39.2 ± 19.5 | 26.0 ± 13.3 | 1.6 ± 1.1 |
| Employment *^a^* | Yes | 35,181 (11.7) | 15.0 ± 11.8 | 21.9 ± 13.4 | 39.1 ± 19.3 | 25.7 ± 13.2 | 1.6 ± 1.1 |
|  | No | 6,851 (2.3) | 14.6 ± 11.5 | 21.6 ± 13.1 | 38.7 ± 19.2 | 26.3 ± 13.4 | 1.7 ± 1.1 |
|  |  |  |  |  |  |  |  |
| Hospitalization > 120 days *^b^* | Yes | 59,667 (19.6) | 14.9 ± 11.6 | 21.7 ± 13.2 | 39.4 ± 19.4 | 25.2 ± 13.0 | 1.6 ± 1.1 |
|  | No | 240,825 (79.0) | 15.4 ± 11.9 | 22.3 ± 13.5 | 39.3 ± 19.5 | 25.9 ± 13.2 | 1.6 ± 1.1 |
|  |  |  |  |  |  |  |  |
| *Pseudopathologies ^c^* |  |  |  |  |  |  |  |
| Thrombosis | Yes | 147,518 (49.3) | 15.1 ± 11.7 | 21.9 ± 13.3 | 39.6 ± 19.4 | 25.2 ± 13.0 | 1.6 ± 1.1 |
|  | No | 151,894 (50.7) | 15.5 ± 12.0 | 22.4 ± 13.6 | 39.0 ± 19.5 | 26.3 ± 13.4 | 1.7 ± 1.1 |
| CVD | Yes | 68,579 (22.9) | 15.2 ± 11.9 | 22.1 ± 13.5 | 39.5 ± 19.5 | 25.3 ± 13.1 | 1.6 ± 1.1 |
|  | No | 230,833 (77.1) | 15.3 ± 11.9 | 22.2 ± 13.4 | 39.2 ± 19.4 | 25.9 ± 13.2 | 1.6 ± 1.1 |
| COPD | Yes | 54,391 (18.2) | 15.2 ± 11.9 | 22.1 ± 13.5 | 39.6 ± 19.5 | 25.3 ± 13.1 | 1.6 ± 1.1 |
|  | No | 245,021 (81.8) | 15.3 ± 11.9 | 22.2 ± 13.4 | 39.2 ± 19.4 | 25.8 ± 13.2 | 1.6 ± 1.1 |
| Asthma | Yes | 4,651 (1.6) | 15.0 ± 11.8 | 22.0 ± 13.4 | 39.5 ± 19.4 | 25.5 ± 13.1 | 1.6 ± 1.1 |
|  | No | 294,761 (98.4) | 15.3 ± 11.9 | 22.2 ± 13.4 | 39.3 ± 19.4 | 25.7 ± 13.2 | 1.6 ± 1.1 |
| Diabetes | Yes | 49,288 (16.5) | 15.1 ± 11.8 | 21.9 ± 13.4 | 39.5 ± 19.3 | 25.3 ± 13.1 | 1.6 ± 1.1 |
|  | No | 250,124 (83.5) | 15.3 ± 11.9 | 22.2 ± 13.4 | 39.3 ± 19.5 | 25.8 ± 13.2 | 1.6 ± 1.1 |
| Psychoses | Yes | 19,255 (6.4) | 15.1 ± 11.8 | 21.8 ± 13.3 | 40.0 ± 19.6 | 25.0 ± 12.8 | 1.6 ± 1.1 |
|  | No | 280,157 (93.6) | 15.3 ± 11.9 | 22.2 ± 13.4 | 39.2 ± 19.4 | 25.8 ± 13.2 | 1.6 ± 1.1 |
| Thyroid affections | Yes | 25,512 (8.5) | 14.7 ± 11.7 | 21.5 ± 13.4 | 39.9 ± 19.4 | 25.1 ± 12.9 | 1.6 ± 1.1 |
|  | No | 273,900 (91.5) | 15.3 ± 11.9 | 22.2 ± 13.4 | 39.2 ± 19.4 | 25.8 ± 13.2 | 1.6 ± 1.1 |
|  |  |  |  |  |  |  |  |
| *Residential environment* |  |  |  |  |  |  |  |
| Population density | Low | 97,259(33.4) | 14.2 ± 11.6 | 20.4 ± 12.9 | 42.1 ± 19.2 | 20.3 ± 10.9 | 1.3 ± 0.9 |
|  | Medium | 105,724 (34.7) | 14.8 ± 11.8 | 21.8 ± 13.3 | 40.0 ± 19.2 | 24.4 ± 11.7 | 1.5 ± 1.0 |
|  | High | 101,771 (33.4) | 16.8 ± 12.1 | 24.2 ± 13.8 | 35.9 ± 19.3 | 32.4 ± 13.9 | 2.0 ± 1.3 |
| Built-up area *^d^* | Low | 99,247 (32.8) | 14.2 ± 11.5 | 20.5 ± 12.8 | 41.8 ± 19.2 | 21.3 ± 11.2 | 1.3 ± 0.9 |
|  | Medium | 102,740 (34.0) | 14.8 ± 11.7 | 21.6 ± 13.1 | 40.1 ± 19.3 | 24.0 ± 12.0 | 1.5 ± 1.0 |
|  | High | 100,313 (33.2) | 16.9 ± 12.3 | 24.3 ± 14.0 | 36.1 ± 19.4 | 31.8 ± 13.8 | 2.0 ± 1.3 |
|  |  |  |  |  |  |  |  |
| Season | Cold | 187,318 (61.5) | 18.1 ± 13.3 | 24.8 ± 15.2 | 32.9 ± 18.1 | 29.1 ± 13.7 | 1.8 ± 1.2 |
|  | Warm | 117,436 (38.5) | 10.9 ± 7.2 | 18.1 ± 8.4 | 49.4 ± 17.1 | 20.5 ± 10.4 | 1.3 ± 0.8 |

n: number of deaths ; sd: standard deviation ; CVD: cardiovascular diseases ; COPD: chronic obstructive pulmonary ; BC: Black carbon

*^a^* in people < 65 years (n=42,032)

*^b^* in the past 12 months preceding death

*^c^* in people > 20 years (n=299,412)

*^d^* available for 302,300 subjects

Air pollutants estimated at the address of the residence the day of death (case day)

# Table S5. I-square statistic and p-values for Cochran Q-test for heterogeneity

|  |  |  | PM_2.5_ | PM_10_ | O_3_ | NO_2_ | Black carbon |
| --- | --- | --- | --- | --- | --- | --- | --- |
| I square-statistic |  |  | 6.8% | 0.0% | 48.9% | 18.2% | 1.4% |
| p-values for Cochran Q-test for heterogeneity |  |  | 0.36 | 0.77 | 0.001 | 0.18 | 0.44 |

# Table S6. Pearson correlations between pollutants

|  |  |  | PM_2.5_ | PM_10_ | O_3_ | NO_2_ | Black carbon |
| --- | --- | --- | --- | --- | --- | --- | --- |
| PM_2.5_ |  |  | 1 | 0.96 | -0.42 | 0.61 | 0.66 |
| PM_10_ |  |  | 0.96 | 1 | -0.35 | 0.64 | 0.67 |
| O_3_ |  |  | -0.42 | -0.35 | 1 | -0.58 | -0.57 |
| NO_2_ |  |  | 0.61 | 0.64 | -0.58 | 1 | 0.84 |
| Black carbon |  |  | 0.66 | 0.67 | -0.57 | 0.84 | 1 |

# Table S7. Percentage changes and 95% confidence intervals associated with 10 μg/m^3^ increase in PM_2.5_, PM_10_, O_3_, NO_2_ and black carbon, 2010-2015 in sensitivity analyses.

|  | PM_2.5_ | PM_10_ | O_3_ | NO_2_ | Black carbon |
| --- | --- | --- | --- | --- | --- |
| Air pollutants variables |  |  |  |  |  |
| Cumulated lags 0 to 1 days | -0.2 (-0.7,0.2) | -0.2 (-0.6,0.2) | 0.3 (-0.2,0.8) | 0.3 (-0.2,0.8) | 0,5 (-7.1,8.8) |
| Cumulated lags 0 to 5 days | 0.4 (0.1,0.9)* | 0.3 (0.0,0.7)* | -0.3 (-0.6,0.0) | 0.6 (0.2,1.1)* | 1.1 (-3.8,6.2) |
|  |  |  |  |  |  |
| Meteorological variables |  |  |  |  |  |
| 6 df instead of 3 df | 0.6 (0.2,1.0)* | 0.4 (0.1,0.8)* | 0.5 (-0.2,1.1) | 1.0 (0.3,1.7)* | 7.0 (-0.3,14.7) |
| 0-1 MA instead of 0-3 MA | 0.7 (0.3,1.1)* | 0.5 (0.2,0.9)* | 0.3 (-0.4,0.9) | 1.1 (0.4,1.8)* | 8.1 (1.2,15.4)* |

n: number of deaths; MA: moving averages

* : significant percentage increases at 5% level


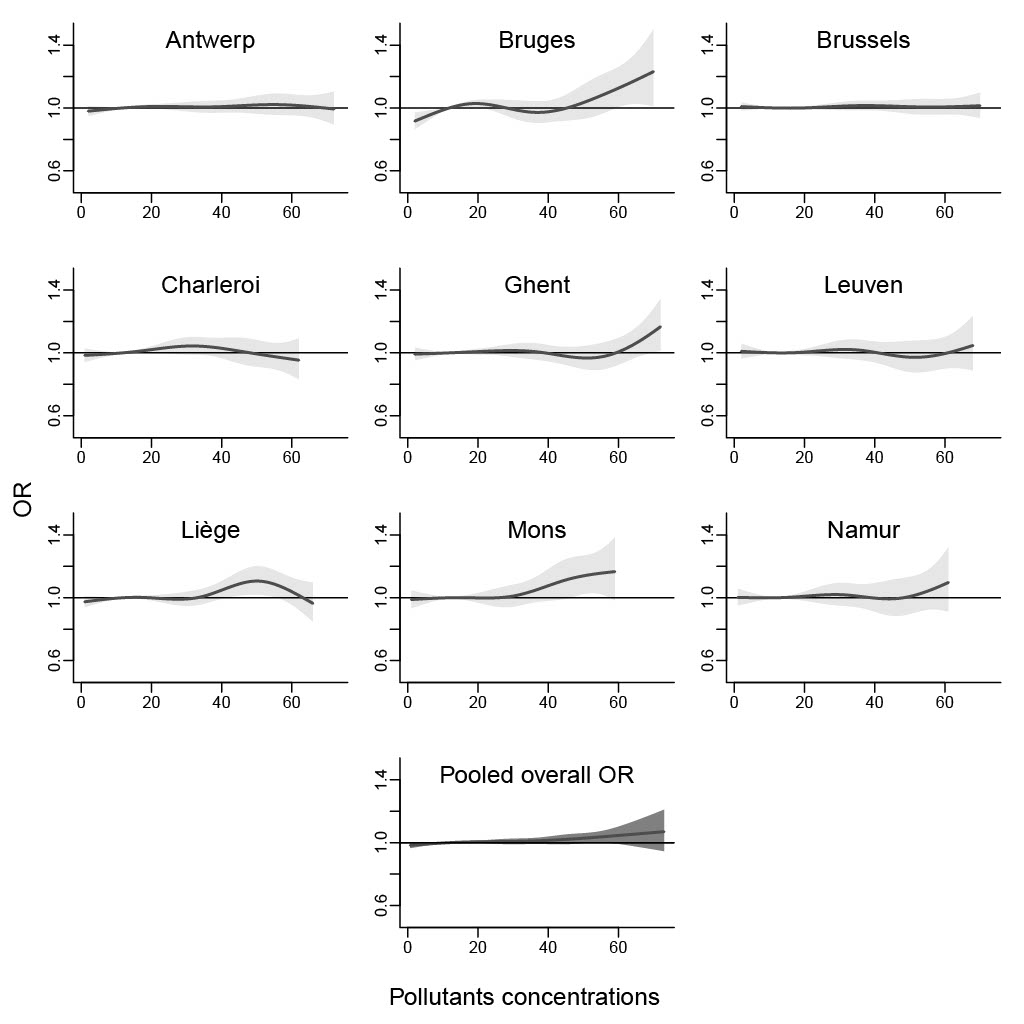


# Figure S1. Agglomeration-specific and pooled PM_2.5_-mortality relationships

Pollutant concentrations in μg/m^3^


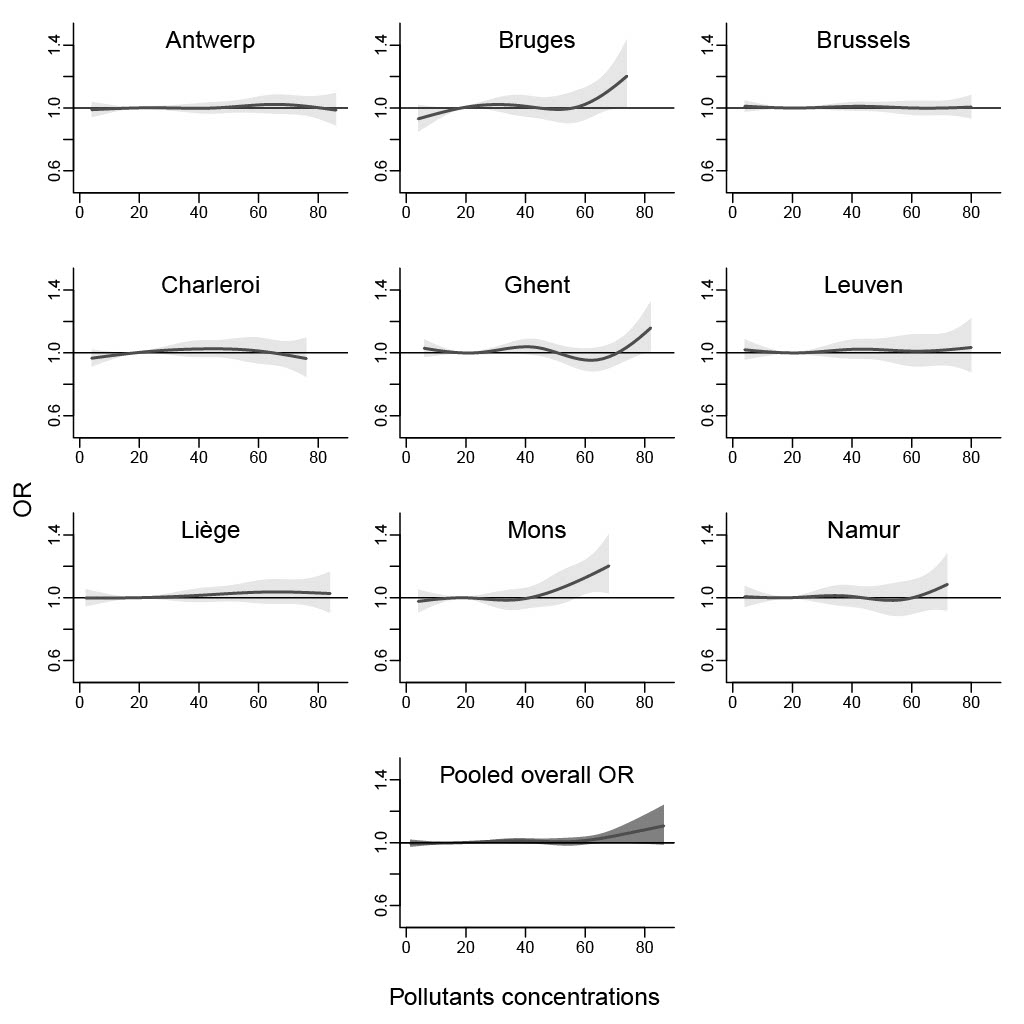


# Figure S2. Agglomeration-specific and pooled PM_10_-mortality relationships

Pollutant concentrations in μg/m^3^


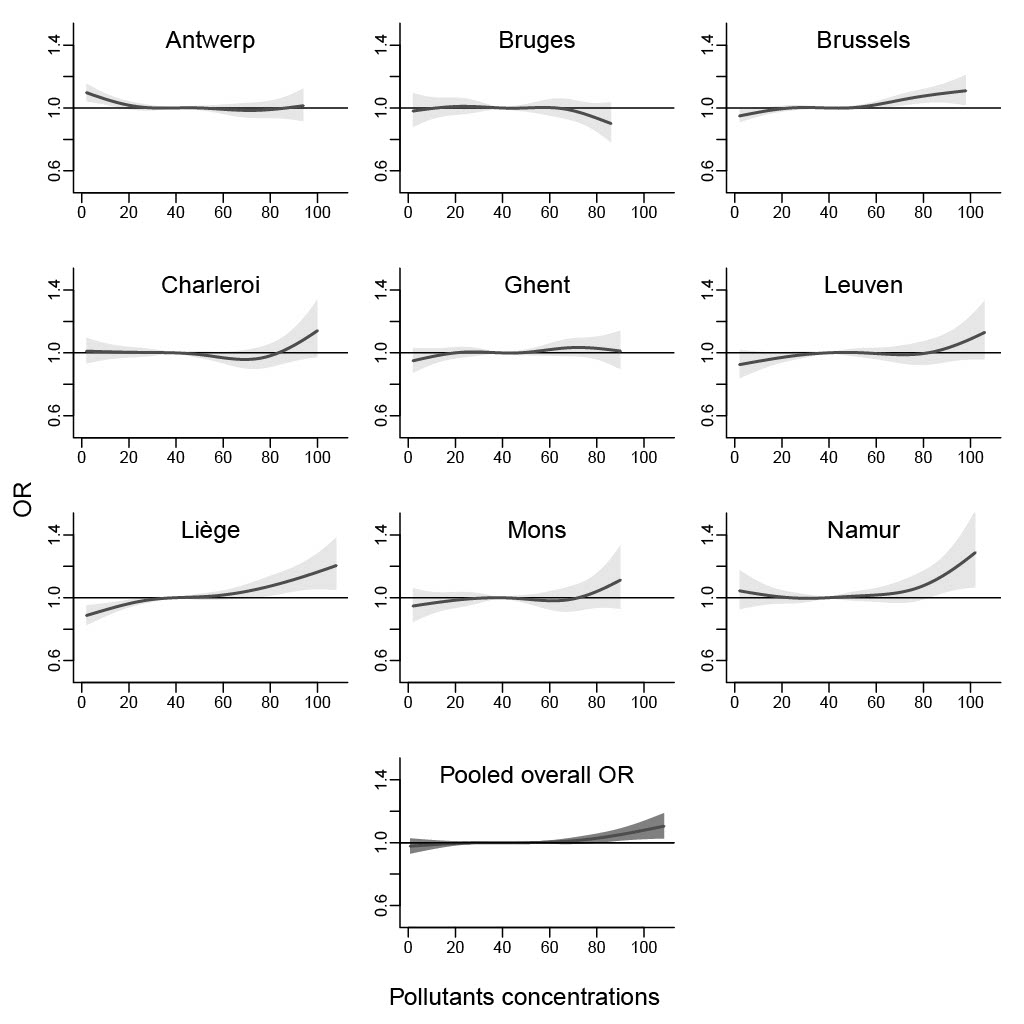


# Figure S3. Agglomeration-specific and pooled O_3_-mortality relationships

Pollutant concentrations in μg/m^3^


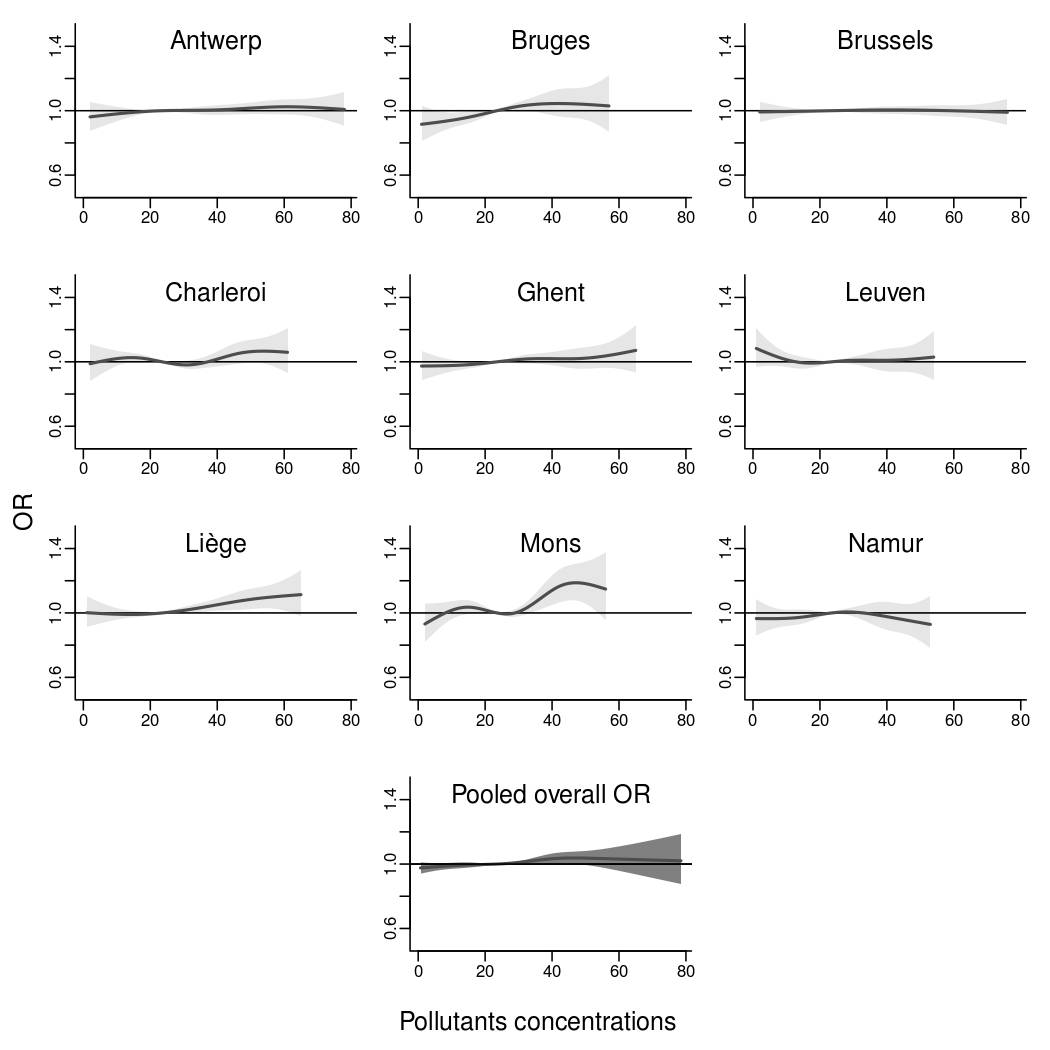


# Figure S4. Agglomeration-specific and pooled NO_2_ -mortality relationships

Pollutant concentrations in μg/m^3^


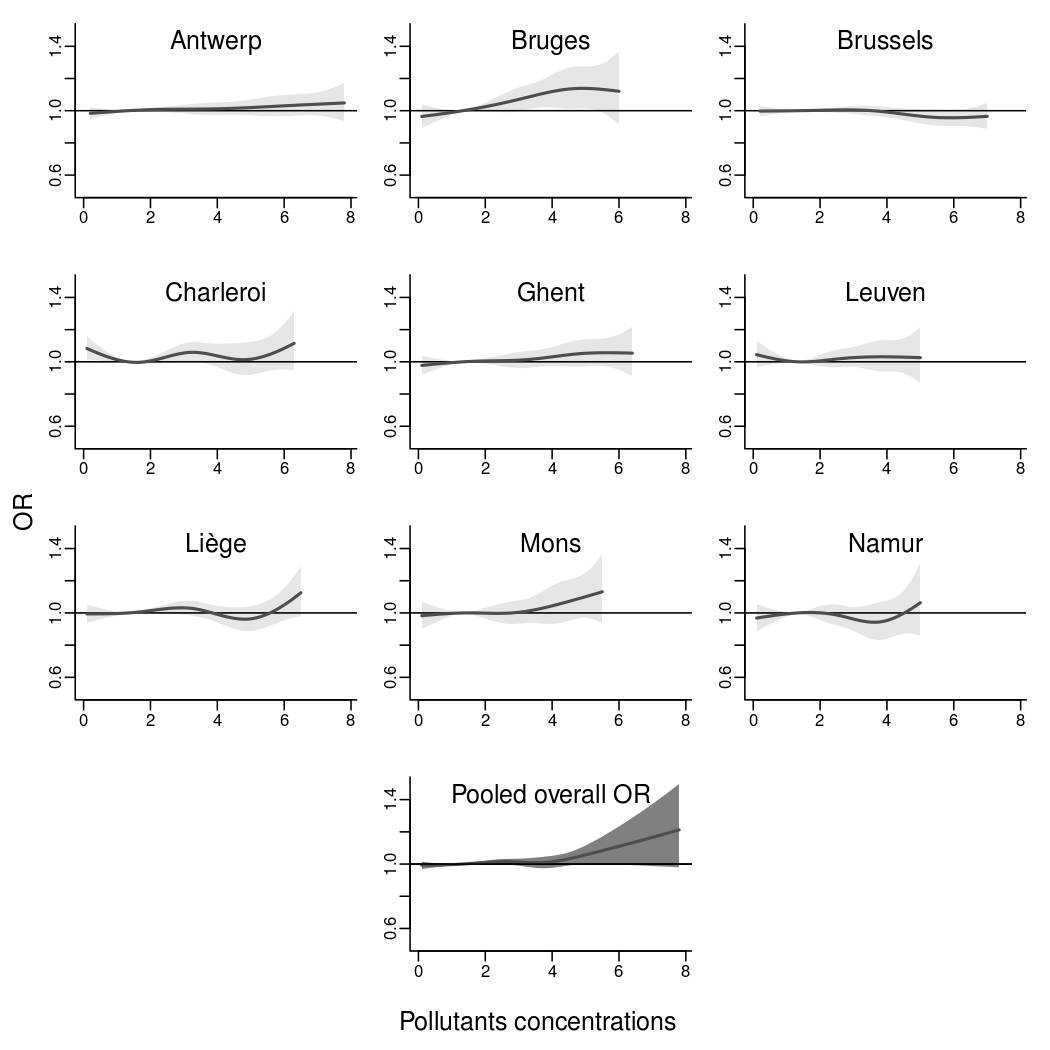


# Figure S5. Agglomeration-specific and pooled black carbon-mortality relationships

Pollutant concentrations in μg/m^3^
